# Cefotaxime Versus Ceftriaxone: A Comprehensive Comparative Review

**DOI:** 10.7759/cureus.69146

**Published:** 2024-09-11

**Authors:** Birendra Sharma, Raghuvendra Chalikwar, Sagar Bhalerao, Ajitkumar A Gondane, Dattatray Pawar, Akhilesh Sharma

**Affiliations:** 1 Surgery, Deepak Hospital, Patna, Bihar, IND; 2 Neurosurgery, Apeksha Hospital, Nanded, IND; 3 Surgical Oncology, Ruby Hall Clinic, Pune, IND; 4 Medical Affairs, Alkem Laboratories Limited, Mumbai, IND

**Keywords:** cefotaxime, ceftriaxone, efficacy, pharmacokinetics, safety

## Abstract

Cefotaxime and ceftriaxone are two prominent third-generation cephalosporin antibiotics, which are a class of antimicrobial agents with overlapping antibacterial spectra and therapeutic indications, commonly used in treating severe bacterial infections, including meningitis, sepsis, and respiratory tract infections. Despite their shared antibacterial coverage, these antibiotics differ significantly in their pharmacokinetic characteristics such as half-life, protein binding, and tissue penetration. This comprehensive review systematically compares the pharmacokinetic profiles, pharmacological attributes, clinical efficacy, and safety profiles of cefotaxime and ceftriaxone. It further highlights the importance of understanding the nuanced differences between cefotaxime and ceftriaxone, particularly in clinical settings such as intensive care units or during pediatric treatment, where rapid bactericidal action or prolonged drug activity might influence therapeutic outcomes. While their overlapping spectrums offer versatility, clinicians should consider these distinct pharmacokinetic attributes and associated clinical outcomes to guide optimal antibiotic selection.

## Introduction and background

Cefotaxime, a third-generation cephalosporin antibiotic approved by the FDA in 1976, effectively targets a broad range of bacteria, including gram-positive and gram-negative species, and anaerobic strains [1,2]. Its versatile antibacterial properties render it valuable for addressing infections caused by susceptible bacterial strains in various anatomical systems, including the lower respiratory tract, genitourinary tract, central nervous system, intra-abdominal regions, bone and joint structures, skin, gynecological structures, septicemia, and as a prophylactic measure for post-surgical infections [3].

Cefotaxime is highly effective against *Enterobacteriaceae*, including multi-drug-resistant strains, but it is less effective against *Pseudomonas aeruginosa* infections [4]. It is not recommended as a standalone treatment for the latter. Intramuscular cefotaxime is successful in treating sexually transmitted infections caused by *Neisseria gonorrhoeae*, and it's valuable for lower respiratory tract pneumonia and complex urinary tract infections [5].

Compared to other cephalosporins, cefotaxime has a low risk of causing coagulopathies and pseudo-cholelithiasis [5]. Clinical trials found similar effectiveness to ceftriaxone [5], with a high-resolution rate (75-100%) for moderate to severe infections [5,6]. It can also be used interchangeably with ceftriaxone off-label to treat endocarditis caused by HACEK (*Haemophilus parainfluenzae*, *Haemophilus aphrophilus*, *Actinobacillus actinomycetemcomitans*, *Cardiobacterium hominis*, *Eikenella corrodens,* and *Kingella kingae*) organisms [7]. When administered intravenously, cefotaxime can cross the blood-brain barrier and treat gram-negative infections resistant to earlier cephalosporins [8].

Ceftriaxone sodium, a third-generation cephalosporin synthesized by F. Hoffmann-La Roche AG (Basel, Switzerland) in 1978, is known for once-daily dosing, making it suitable for both intravenous and intramuscular administration in outpatient settings and strong resistance to β-lactamases, making it widely used over first and second generation cephalosporins [9]. Ceftriaxone is a prominent antibiotic known for its potent antibacterial properties, broad-spectrum activity, and low toxicity risk. It is used to treat various bacterial infections like bronchitis, pneumonia, osteomyelitis, and more. Common side effects are similar to other beta-lactam antibiotics, but prolonged or high-dose use can lead to biliary pseudolithiasis [10-12].

Despite their similar therapeutic applications, healthcare professionals frequently encounter difficulties when choosing between cefotaxime and ceftriaxone due to their distinct pharmacokinetic properties, variations in clinical efficacy, safety profiles, and differences in susceptibility patterns. This comparative review is especially relevant in light of the evolving landscape of bacterial resistance, characterized by the emergence of extended-spectrum β-lactamase (ESBL)-producing organisms and multidrug-resistant pathogens. Reassessing the roles of these antibiotics is crucial to optimizing empirical therapy, enhancing patient outcomes, and mitigating the development of antimicrobial resistance.

## Review

Methodology

Literature Search Strategy

**Figure 1:**
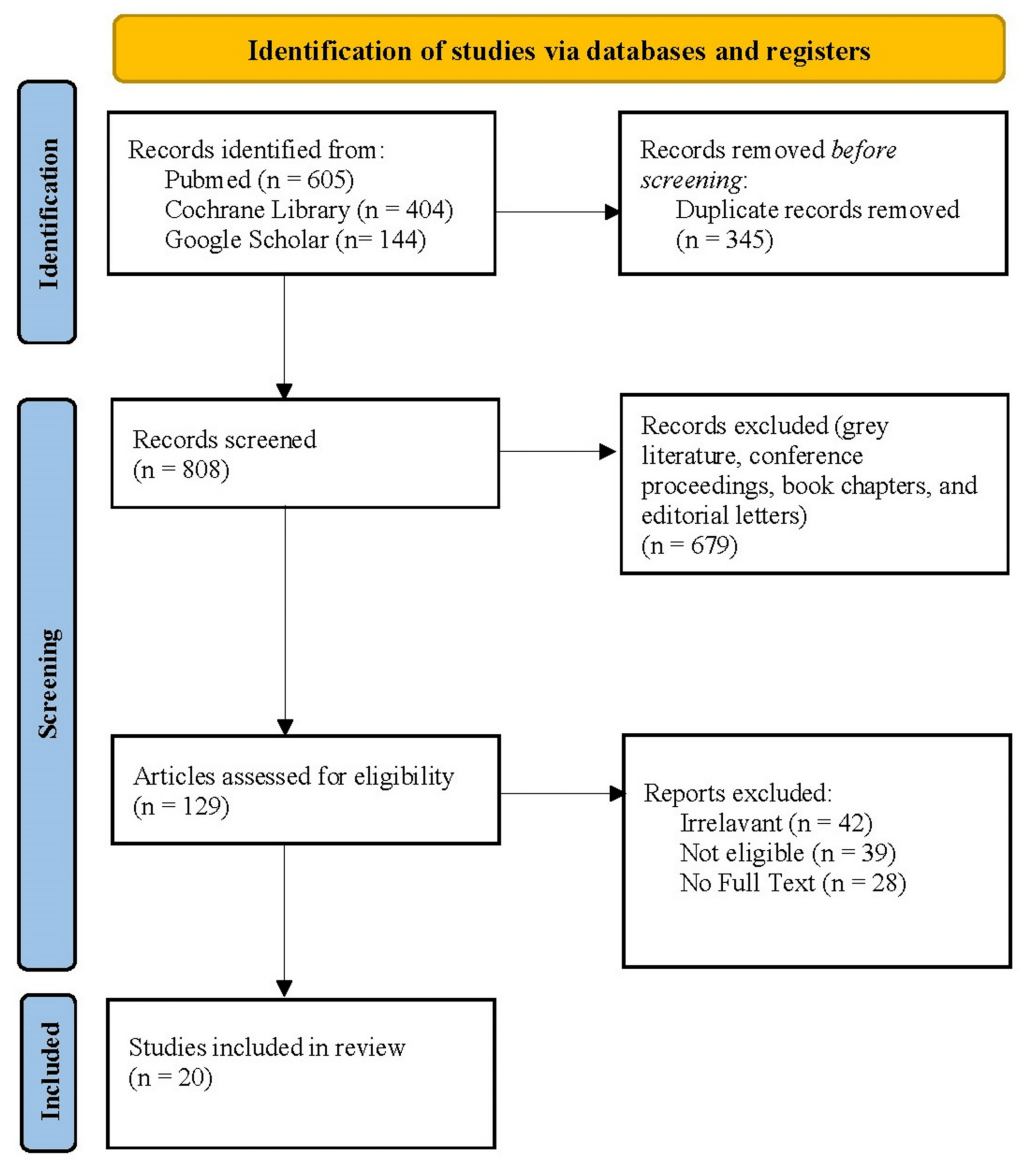
PRISMA flowchart depicting the selection of the studies. Studies were identified from PubMed (n = 605), Cochrane Library (n = 404), and Google Scholar (n = 114), with duplicate records (n = 345) removed. After screening 808 records, 679 were excluded based on criteria such as grey literature, conference proceedings, and editorial letters. Out of 129 eligible studies, 109 were excluded due to irrelevance (n = 42), ineligibility (n = 39), or lack of full text (n = 28), resulting in 20 studies that were included in the present review. PRISMA: Preferred Reporting Items for Systematic Reviews and Meta-Analyses

A systematic review was conducted using a comprehensive and methodologically rigorous search strategy to compare cefotaxime and ceftriaxone in terms of efficacy, safety, and pharmacokinetics. The search encompassed several key databases, including PubMed, Cochrane Library, and Google Scholar. PubMed was selected as a primary source due to its extensive coverage of biomedical literature, while the Cochrane Library was searched to identify systematic reviews, and clinical trials pertinent to the comparison, and Google Scholar was utilized to access grey literature and additional relevant sources not indexed in traditional databases (as shown in Figure 1).

The search was performed using a combination of carefully chosen keywords and search terms to ensure thorough retrieval of relevant studies. These search terms used included "Cefotaxime AND Ceftriaxone," "Cefotaxime OR Ceftriaxone AND Comparative efficacy OR Clinical trials," "Cefotaxime OR Ceftriaxone AND Pharmacokinetics AND Safety profile," "Cefotaxime OR Ceftriaxone AND In vitro susceptibility OR Zone of Inhibition," "Cefotaxime OR Ceftriaxone AND Side effects OR Tolerability," and "Cefotaxime OR Ceftriaxone AND Nephrolithiasis AND Cholelithiasis." This search approach was designed to capture the full scope of the literature related to the clinical and pharmacokinetic properties of these two antibiotics. The search was limited to articles published in English up to December 2023.

Study Selection Criteria

Studies were included based on several criteria: only those studies directly comparing cefotaxime and ceftriaxone in terms of efficacy, safety, and pharmacokinetics were considered. The present review focused on clinical trials and observational studies published in peer-reviewed journals, with a restriction to English-language publications to maintain consistency. Both adult and paediatric populations were included to provide a comprehensive overview across different age groups. Exclusion criteria were strictly applied to maintain the review's focus and relevance. Studies not directly comparing the two antibiotics, non-English language publications, and those lacking sufficient data, such as case reports or letters to the editor, were excluded. Additionally, articles that were not available in full text were omitted to ensure a thorough assessment of the study methodologies and results.

Study Quality and Bias Mitigation

To ensure the reliability and validity of this review, we conducted a comprehensive assessment of the included studies’ quality, and potential biases. Each study was critically appraised using standardized tools, and only those meeting stringent criteria for methodological rigor were included. Potential biases, such as selection, performance, detection, and reporting biases, were carefully considered and addressed through a thorough evaluation process. The incorporation of these quality control measures minimizes the risk of bias, thereby enhancing the credibility of the comparative analysis of cefotaxime and ceftriaxone presented in this review.

Data Extraction and Synthesis

The screening process involved three stages to ensure the selection of high-quality studies. Initially, articles were screened based on titles and abstracts to exclude irrelevant studies. The remaining articles underwent a full-text review to assess their eligibility based on the predefined criteria. Finally, data extraction was performed on the included studies, focusing on key outcomes such as efficacy, safety profiles, pharmacokinetics, and in vitro susceptibility. This systematic approach provided a robust and unbiased comparison of cefotaxime and ceftriaxone, ensuring a scientifically rigorous evaluation of the available literature.

Results

Pharmacokinetic Properties

Cefotaxime is recommended in a dose of 0.5 to up to 12 grams per day (in two to four divided doses) depending on the type of infection while ceftriaxone is recommended to be used in a daily dose of 1-2 grams given once a day (or in equally divided doses twice a day) depending on the type and severity of infection (Maximum dose should not exceed 4 grams). Although, both cefotaxime and ceftriaxone are recommended to be used via parenteral routes only, they diverge significantly in terms of serum protein binding and elimination half-life. The comparative pharmacokinetics are described in Table 1 [13,14]. Serum protein binding of an antibiotic is one of the major factors determining its extravascular penetration in peripheral human lymph nodes. As the level of protein binding increases, the penetration ratios of extravascular and serum levels gradually decrease. The high serum protein binding of ceftriaxone could impact its therapeutic efficacy as compared to cefotaxime with much lesser protein binding [15-17].

**Table 1:** Pharmacokinetic comparison of cefotaxime and ceftriaxone. This table presents the pharmacokinetic parameters of cefotaxime and ceftriaxone, including plasma protein binding, volume of distribution (Vd), metabolism, clearance, and elimination half-life (t1/2). Cefotaxime shows lower plasma protein binding (35% vs 95%) and a shorter elimination half-life (1.2 vs 8.8 h) compared to ceftriaxone. However, cefotaxime shows a higher Vd (14 vs 10 L) than ceftriaxone. The metabolism and clearance routes differ significantly, with cefotaxime being primarily metabolized in the liver and cleared renally, while ceftriaxone undergoes major metabolism in the intestine with cleared majorly through the urine [13,14].

Parameters	Cefotaxime	Ceftriaxone
Plasma Protein Binding	35%	95%
Volume of distribution (L)	14	10
Metabolism	Liver	Intestine
Clearance	Renal	Major – Liver; Minor – Urine
Elimination t_1/2_	1.2 hours	8.8 hours

These pharmacokinetic differences significantly influence dosing strategies in specific patient populations. For instance, in patients with renal impairment, cefotaxime, which is primarily cleared by the kidneys, may require dose adjustments to prevent accumulation and toxicity. Conversely, ceftriaxone, with its dual elimination pathway involving both the liver and the kidneys, might be preferred in patients with renal dysfunction but should be used cautiously in those with hepatic impairment. Similarly, in patients with severe infections such as pneumonia, the choice of antibiotic and dosing frequency may be influenced by these pharmacokinetic characteristics. Although ceftriaxone is recommended for once-daily use as per elimination half-life, a once-daily ceftriaxone regimen exhibited a high failure rate (39%) in one study [18], suggesting it may not be adequate for treating severe pneumonia in critically ill patients [17]. Further, in contrast, a study indicated that thrice-daily cefotaxime and twice-daily dosing were equally effective [19]. In another hospital-based retrospective review, no difference was observed in patient outcomes between 12-hour and eight-hour cefotaxime regimens, supported by pharmacokinetic data showing that 12-hour dosing is sufficient for seriously ill patients [20].

In vitro Susceptibility and Effectiveness

Both cefotaxime and ceftriaxone have shown activity against a wide spectrum of gram-positive, gram-negative, and anaerobic bacteria. The list of susceptible bacteria is shown in Table 2.

**Table 2:** List of bacteria susceptible to cefotaxime and ceftriaxone. References: [21,22]

Bacteria Susceptible to Cefotaxime	Bacteria Susceptible to Ceftriaxone
Gram-Positive Bacteria	
*Enterococcus* spp.	Staphylococcus aureus
*Staphylococcus aureus* (methicillin-susceptible isolates only)	Staphylococcus epidermidis
Staphylococcus epidermidis	Streptococcus pneumoniae
Streptococcus pneumoniae	Streptococcus pyogenes
*Streptococcus pyogenes *(Group A beta-hemolytic streptococci)	Viridans group streptococci
*Streptococcus* spp. (Viridans group streptococci)	
Gram-Negative Bacteria	
*Acinetobacter* spp.	Acinetobacter calcoaceticus
*Enterobacter* spp.	Enterobacter aerogenes
Escherichia coli	Enterobacter cloacae
Haemophilus influenzae	Escherichia coli
Haemophilus parainfluenzae	Haemophilus influenzae
*Klebsiella* spp.(including* Klebsiella pneumoniae)*	Haemophilus parainfluenzae
Morganella morganii	Klebsiella oxytoca
*Neisseria gonorrhoeae* (including beta-lactamase-positive and negative strains)	Klebsiella pneumoniae
Neisseria meningitidis	Moraxella catarrhalis
Proteus mirabilis	Morganella morganii
Proteus vulgaris	Neisseria gonorrhoeae
Providencia rettgeri	Neisseria meningitidis
Providencia stuartii	Proteus mirabilis
Serratia marcescens	Proteus vulgaris
*Citrobacter* spp.	Pseudomonas aeruginosa
	Serratia marcescens
Anaerobic bacteria	
*Bacteroides* spp. (including some isolates of *Bacteroides fragilis*)	Bacteroides fragilis
Clostridium spp. (most isolates of *Clostridium difficile *are resistant)	*Clostridium* spp.
*Fusobacterium* spp. (including Fusobacterium nucleatum)	*Peptostreptococcus *spp.
Peptococcus spp.	
*Peptostreptococcus* spp.	

The susceptibility of bacteria to these antibiotics varies geographically and changes over time, therefore local susceptibility plays an important role in deciding the antibiotic of choice. Gondane et al. conducted a comparative in vitro susceptibility study of cefotaxime and ceftriaxone on clinical bacterial isolates collected from various regions in India [23]. They tested clinical samples with positive bacterial cultures using E-test strips and disk diffusion methods to determine minimum inhibitory concentrations (MIC) and zones of inhibition (ZOI). Interpretations followed Clinical and Laboratory Standards Institute (CLSI) guidelines to assess antibiotic effectiveness. Among 400 clinical isolates, *Escherichia coli *was the most common (47.75%), followed by *Klebsiella* (26%), *Salmonella* (7.75%), *Proteus* (3.8%), and *Acinetobacter* (2.8%). The mean MIC values for *E. coli*, *Klebsiella, Staphylococcus, Citrobacter koseri*, and *Serratia marcescens *were found to be comparatively lower with cefotaxime as compared to ceftriaxone. However, this variance did not achieve statistical significance. Nevertheless, cefotaxime demonstrated a significant increase in the Zone of Inhibition (ZOI) compared to ceftriaxone for *E. coli*, *Klebsiella*, and *Salmonella*. They concluded that cefotaxime exhibits a more favorable sensitivity profile in terms of both MIC and ZOI when compared to ceftriaxone, thus rendering it a preferable choice for the empirical treatment of patients suffering from infections caused by these isolated organisms. A similar study conducted by Gondane et al. involved in vitro comparison of cefotaxime-sulbactam and ceftriaxone-sulbactam on 400 clinical samples originating from urinary tract infections (42.75%), lower respiratory tract infections (20.75%), skin and soft tissue infections (16.75%), and sepsis (12.75%). Notably, the cefotaxime-sulbactam group showed significantly higher ZOI for *E. coli, Klebsiella*, and *Salmonella* compared to the ceftriaxone-sulbactam group. Additionally, MIC50 values for *E. coli *and *Klebsiella* were lower in the cefotaxime-sulbactam group than in the ceftriaxone-sulbactam group, indicating better sensitivity. This study suggests that cefotaxime-sulbactam may be more effective against these bacterial isolates in the Indian context [24].

These in vitro findings suggest potential clinical relevance, particularly in the face of increasing antimicrobial resistance. While in vitro susceptibility does not always translate directly to clinical success, higher sensitivity profiles, such as those demonstrated by cefotaxime and its combination with sulbactam, may offer a therapeutic advantage in empirical treatment, especially in regions with prevalent resistant organisms. Understanding these patterns can help clinicians make informed choices that align with local resistance trends and improve patient outcomes.

Clinical Efficacy and Safety

Clinical evidence of efficacy in treatment: Cefotaxime and ceftriaxone have similar clinical indications and thus have been compared in various clinical studies. A prospective, randomized, single-blind, comparative study was conducted by Reeves et al. with the objective of evaluating the effectiveness of cefotaxime in comparison to ceftriaxone for the treatment of severe chest infections [17]. Their study included 51 patients who were randomly divided into two groups. Both groups had similar demographic characteristics, allowing for a fair comparison of the two drugs. One group received ceftriaxone at a dose of 2 grams administered intravenously once daily, while the other group received cefotaxime at a dose of 2 grams administered intravenously thrice daily. Both treatment regimens were administered over a five-day period. The two patient groups had similar demographic characteristics. Notably, it was observed that administering ceftriaxone at a daily dose of 2 grams may not yield satisfactory therapeutic results in the treatment of severe chest infections, indicating the superiority of cefotaxime over ceftriaxone.

Another multicenter study conducted by Shah and Stille aimed to compare the effectiveness of cefotaxime and ceftriaxone in the treatment of nosocomial pneumonia, a total of 56 patients were randomized in the cefotaxime group, and 62 patients in the ceftriaxone group [20]. Their study results indicate that both treatment regimens, namely, administering 2 grams of cefotaxime every 12 hours and 4 grams of ceftriaxone once daily or 2 grams every 12 hours, demonstrated efficacy in managing nosocomial pneumonia. While both antibiotics demonstrated equivalent efficacy in managing nosocomial pneumonia, differences in patient populations across centers, including variability in local resistance patterns and clinical management practices, could influence the study outcomes and limit the direct comparability of results.

Thomas et al. conducted a prospective randomized double-blind trial to assess the comparative efficacy and safety of cefotaxime and ceftriaxone in intensive care unit (ICU) patients afflicted with severe infections necessitating systemic antimicrobial therapy [25]. Patients were subjected to random allocation into two groups: one receiving cefotaxime at a dosage of 1 gram intravenously thrice daily and the other receiving ceftriaxone at a dosage of 2 grams every 24 hours. Upon completion of the treatment regimen, an analysis utilizing contemporary statistical methods revealed that 67% of patients treated with cefotaxime and ceftriaxone demonstrated clinical recovery or improvement. Notably, bacteriologic responses appeared more favorable in the cefotaxime group, with a rate of 55% compared to 42% in the ceftriaxone group. Based on these preliminary findings, it is reasonable to conclude that, at the prescribed dosages employed in this study, cefotaxime and ceftriaxone exhibit equivalent efficacy in the management of infections encountered in the ICU setting.

Smith et. al in another study compared ceftriaxone to cefotaxime in serious bacterial infection, the results showed that both drugs were effective and well tolerated [26]. The only empiric difference of note was the finding that all three enterococcal urinary tract superinfections occurred in the ceftriaxone group. This difference may be attributable to cefotaxime's marginal in vitro activity against enterococci, a property not shared by ceftriaxone [27].

The efficacy and safety of cefotaxime and ceftriaxone have also been compared in the management of pregnancy with preterm premature rupture of membranes. In a retrospective data analysis, Rasti et al. noted that the most commonly used therapeutic regimens involved administering cefotaxime at 3 grams once or ceftriaxone 2 grams once [28]. No significant differences were observed in the latency period and infant outcomes, including birth weight and Apgar scores, between the use of cefotaxime and ceftriaxone. However, cefotaxime appeared to extend the latency period for more than 48 hours, enhancing the prospects for fetal lung maturation and proving to be more cost-effective than ceftriaxone.

In clinical investigations, comparative trials have consistently indicated that cefotaxime and the third-generation cephalosporin ceftriaxone exhibit equivalent clinical efficacy [5]. Furthermore, in the context of hospitalized patients suffering from moderate to severe infections, studies have reported notably high-resolution rates, ranging from 75% to 100% [5,6]. Importantly, cefotaxime exhibits the capability to effectively traverse the blood-brain barrier following intravenous administration, enabling its utility in managing gram-negative infections that exhibit resistance to earlier generations of cephalosporins [8]. Cefotaxime is a safe and effective agent in the treatment of gram-negative enteric bacillary meningitis in infants and children and should be considered as a potential drug of choice in gram-negative neonatal meningitis due to susceptible organisms [29]. 

Besides these comparative studies, cefotaxime has individually been assessed for various other indications and found to be useful. Takase studied the utilization of cefotaxime in the context of gynecological and obstetrical infections [30]. Their study assessed the penetration of cefotaxime into human uterine tissue following a single intravenous injection of 1 gram. A peak concentration of 12.24 micrograms per gram was achieved within approximately 20 minutes. Furthermore, the study explored the placental transfer of cefotaxime after a single 1-gram intravenous injection. Notably, when analyzing concentrations in the neonate's serum, it was found to be approximately one-fourth of that in the mother's serum, while amniotic fluid concentrations were roughly one-tenth. The maximum concentration observed in the neonate was 0.63 micrograms per milliliter. The clinical response rates were highly favorable, with success rates of 98% against uterine infections, 94% against adnexitis, 91% against pelvic infections, and 93% against external genital infections. Bacteriological effectiveness, assessed by pathogen type, was also notable, demonstrating eradication rates of 95% against gram-positive aerobes, 88% against gram-negative aerobes, and 96% against gram-negative anaerobes.

In a multicentre open study carried out by Hemsell et al., cefotaxime was administered for the treatment of endometritis following cesarean section, pelvic cellulitis post-hysterectomy, and acute pelvic inflammatory disease [31]. The drug achieved clinical remission in 93% of 104 women. In a randomized comparative study conducted at a single center, cefotaxime resolved endometritis in 97% of 36 cases following cesarean section, while a combination of clindamycin and gentamicin achieved remission in 94% of 18 cases with the same infection. Importantly, no significant perturbations in hematopoietic, hepatic, or renal function were observed with either treatment regimen. These findings suggest that cefotaxime is a safe and exceptionally effective antimicrobial agent, ideally suited for monotherapy in the treatment of severe soft-tissue pelvic infections among obstetric or gynecologic patients.

Several studies have reported varying rates of bacteriological cure with cefotaxime. Runyon et al. observed a bacteriological cure rate of 93.1% in patients with spontaneous bacterial peritonitis [32]. Kaur et al. documented a rate of 93.3% in urinary tract infection patients when cefotaxime was combined with sulbactam [33]. Chen et al. reported a cure rate of 79.1% in patients of spontaneous bacterial peritonitis with cirrhosis [34], while Cordero et al. found a bacteriological cure rate of 93.4% in HIV-infected patients with bacterial pneumonia treated with cefotaxime [35].

Perkins conducted clinical trials assessing the efficacy of cefotaxime in the management of bacterial infections affecting the lower respiratory tract [36]. Upon a comprehensive analysis of patient responses, the comparative studies revealed statistically significant differences solely in the clinical responses to cefotaxime within the context of a single-blind randomized trial (P = 0.03). Notably, favorable cure rates were achieved with cefotaxime in patients afflicted by infections stemming from a spectrum of pathogens, including *S. pneumoniae*, *H. influenzae*, *S. pyogenes*, *S. aureus*, and *E. coli*. Moreover, responses were nearly equivalent for infections caused by *Proteus, Enterobacter*, and *Klebsiella* species. Cefotaxime also demonstrated clinical efficacy in select cases of infections attributed to *S. marcescens *and *P. aeruginosa*. While all *Serratia* strains exhibited high susceptibility to cefotaxime in vitro, the range of minimal inhibitory concentrations for *Pseudomonas* isolates displayed considerable variation.

The details of a few comparative clinical studies are shown in Table 3.

**Table 3:** Head-to-head comparative studies of cefotaxime versus ceftriaxone.

Author	Indication	Study Design	Number of subjects	Dose Used	Conclusion
Reeves et al., 1989 [17]	Chest infection	Prospective, randomized, single blind	51	Cefotaxime: 2gm IV TID Ceftriaxone: 2gm IV once daily	Administration of ceftriaxone at a single daily dose of 2 grams may not provide a satisfactory therapeutic outcome for the treatment of severe chest infections indicating the advantage of Cefotaxime over ceftriaxone
Shah and Stille, 1995 [20]	Nosocomial pneumonia	Prospective, randomized, multicentre	118	Cefotaxime: 2gm IV BID Ceftriaxone: 2gm IV BID	Both the drugs exhibited equivalent effectiveness in the treatment of nosocomial pneumonia
Thomas et al., 1992 [25]	Intensive care unit (ICU) patients afflicted with severe infections	Prospective, randomized, double blind	34	Cefotaxime: 1gm IV TID Ceftriaxone: 2gm IV OD	Bacteriologic responses appeared more favorable in the cefotaxime group, with a rate of 55% compared to 42% in the ceftriaxone group.
Rasti et al., 2020 [28]	Prolongation of latency period in preterm premature rupture of membranes	Retrospective study	52	Cefotaxime: 3gm OD Ceftriaxone: 2gm OD	Cefotaxime appeared to extend the latency period for more than 48 hours, enhancing the prospects for fetal lung maturation and proving to be more cost-effective than ceftriaxone
Smith et al., 1989 [26]	Serious bacterial infections	Prospective, randomized, double blind	171	Cefotaxime: 2gm every 4 hrs Ceftriaxone: 2gm IV OD	Both drugs were effective and well tolerated. The only empiric difference of note was the finding that all three enterococcal urinary tract superinfections occurred in the ceftriaxone group

Clinical evidence of efficacy in prophylaxis: Mohan et al. conducted a study in which participants received a single intravenous dose of 1 gram of cefotaxime prior to their obstetric and gynecological surgeries [37]. Subsequently, the participants were monitored for the occurrence of postoperative complications, including wound infections and the necessity for wound resuturing. Their study’s conclusion supported the efficacy of single-dose cefotaxime prophylaxis, which was found to be equally effective as conventional multi-dose antibiotic therapy. Furthermore, it was deemed a cost-effective and safe approach for both obstetric and gynecological surgical procedures.

Preterm premature rupture of the membranes is associated with a high risk of neonatal sepsis. An increase in the incidence of early-onset neonatal sepsis due to ampicillin-resistant *E. coli* in premature infants has been observed in the past few years. Intrapartum prophylaxis with ampicillin has proven to be efficient for the prevention of early neonatal sepsis due to group B streptococci. To date, there is no strategy for the prevention of early neonatal sepsis due to ampicillin-resistant *E. coli *[38]. Lepercq et al. conducted a study wherein intravenous administration of cefotaxime during labor followed a specific regimen: an initial 2 grams at the onset of labor, and subsequently, 1 gram every four hours until delivery [38]. This protocol yielded cord blood concentrations of cefotaxime spanning from 0.5 to 8.5 milligrams per liter. Importantly, these concentrations consistently achieved the MIC90 (MIC required to inhibit 90% of bacterial strains) of cefotaxime for *E. coli* strains, which was 0.125 milligrams per liter, in all cases. Their study underscores that prophylactic administration of cefotaxime during delivery in pregnant women harboring ampicillin-resistant isolates within the *Enterobacteriaceae* family can result in cefotaxime concentrations in fetal blood at delivery that surpass the MIC threshold.

Further evidence supports the use of cefotaxime in broader prophylactic applications, including surgical procedures and in hospital settings to prevent hospital-acquired infections. Marano et al. conducted a systematic review demonstrating cefotaxime's efficacy in reducing surgical site infections (SSIs), particularly in high-risk surgeries such as colorectal procedures. The review found that cefotaxime, when used as a single-dose prophylactic antibiotic, effectively minimized the incidence of SSIs with a favorable safety profile, particularly in patients undergoing gastrointestinal and gynecological surgeries​ [39]. Additionally, cefotaxime's role in preventing hospital-acquired infections has been substantiated in surgical ICUs, where its use was associated with a significant reduction in the incidence of ventilator-associated pneumonia and catheter-associated urinary tract infections, due to its potent activity against common ICU pathogens, including *E. coli*, *K. pneumoniae*, and *P. aeruginosa ​*[40]. The collective evidence highlights cefotaxime’s versatility and effectiveness as a prophylactic agent in diverse clinical settings, aligning with current antimicrobial stewardship goals to optimize antibiotic use and reduce hospital-acquired infections.

Clinical evidence of safety and tolerability: Cefotaxime and ceftriaxone, both belonging to the same pharmacological class, share many similarities in their pharmacological properties. However, they diverge in terms of serum protein binding, elimination half-life, and excretion route. Ceftriaxone exhibits higher protein binding (95% vs. 35%), a longer elimination half-life (8.8 hours vs. 1.2 hours), and a greater degree of biliary excretion compared to cefotaxime (40% vs. 10%) [41,42]. These distinctions may contribute to differences in their respective side-effect profiles.

Both cefotaxime and ceftriaxone have generally well-tolerated profiles in adults and children when administered intravenously or intramuscularly. Common adverse effects of cefotaxime include localized reactions at the injection site, with a similar incidence to procaine penicillin G, and phlebitis. Cefotaxime has been associated with rash, diarrhea, and temporary changes in renal and hepatic function test results, but no symptomatic drug-induced kidney or liver issues. Ceftriaxone has a comparable tolerability profile with common adverse effects such as diarrhea, exanthema, rash, pruritus, localized reactions at the injection site, and transient variations in laboratory tests; however, it has additionally also been associated with hematoma, hemorrhage, hypoprothrombinaemia bleeding in chronic dialysis patients, thrombocytopenia, periorbital edema, facial angioedema, and hypokalaemia not seen with ceftriaxone. Notably, neither drug caused symptomatic nephrotoxicity or hepatotoxicity [4,13,43-47].

Discussion

Clostridium difficile and Pseudomonas Infection Secondary to Drug Administration

The majority of cephalosporins, including cefotaxime, cefadroxil, cephalexin, cefuroxime, ceftazidime, and ceftobiprole, primarily undergo renal excretion through glomerular filtration. In contrast, a subset of cephalosporins, exemplified by ceftriaxone, experience substantial biliary excretion, accounting for a significant portion (30%-40%) of their active form [48]. This biliary excretion route leads to a notable presence of the antibiotic substance in the intestines, resulting in a sustained impact on the gut microbiota. The human intestinal microbiome serves as a pivotal host defence mechanism against the development of *C. difficile *infections. The disruption of the gut microbiota can create favorable conditions for the onset of *C. difficile* infections. It is noteworthy that cephalosporins, in general, exhibit limited in vitro activity against *C. difficile* [49].

When substantial quantities of ceftriaxone come into contact with the intestinal microbiota, it can contribute to the selection of *C. difficile*. Furthermore, ceftriaxone has the propensity to stimulate spore germination and toxin production by *C. difficile* [49]. Both of these properties, including the extensive biliary excretion of the antibiotic, appear to exert a significant and augmenting influence on the incidence of *C. difficile* infections. A study by Wendt et al. supports the increased risk of C. difficile infections associated with ceftriaxone use [50]. This risk is linked to ceftriaxone's long-term use, which is also associated with a higher incidence of biliary complications, such as cholelithiasis and biliary sludge, due to its higher protein binding and biliary excretion. These factors can disrupt the gut microbiota and raise susceptibility to infections like *C. difficile*. In contrast, cefotaxime, with its lower biliary excretion and primarily renal clearance, presents a reduced risk profile, making it a potentially safer option for prolonged therapy, especially in patients with liver conditions or at risk for biliary complications [50].

Ceftriaxone has a greater tendency to promote the proliferation of *Candida* spp. in vaginal flora, to induce overgrowth of *Candida *spp. and enterococci in the intestine, and to lead to adverse effects such as diarrhea, urolithiasis, and cholelithiasis in contrast to cefotaxime [51-56]. Ceftriaxone, owing to its significant biliary excretion, can disrupt gut microbiota to a greater extent, whereas cefotaxime has a lower level of biliary excretion than ceftriaxone. A noteworthy observation at Leipzig University Hospital in Germany revealed a reduction in the use of ceftriaxone alongside increased utilization of cefotaxime, resulting in a marked decrease in the incidence of *C. difficile* infections [50].

Ceftriaxone's biliary excretion has been associated with an increase in *Enterobacteriaceae *carrying high-level AmpC β-lactamase (HL-CASE), leading to resistant infections that necessitate the use of carbapenems. A retrospective study conducted in France demonstrated that replacing ceftriaxone with cefotaxime in clinical practice lowered and stabilized the incidence of HL-CASE *Enterobacteriaceae* [57]. Consequently, the use of cefotaxime may contribute to reduced reliance on carbapenems, which are preferred in such scenarios.

Nephrolithiasis and Cholelithiasis

Ceftriaxone has been associated with both, nephrolithiasis and cholelithiasis. In a study conducted by Ustyol et al., which involved a comparative assessment of ceftriaxone- and cefotaxime-induced biliary pseudolithiasis or nephrolithiasis in 154 pediatric subjects, it was observed that abnormal biliary sonographic findings were present in significantly higher (P=0.006) proportion of children treated with ceftriaxone (20.9%) as compared to those treated with cefotaxime (5.9%) [58]. Among 20.9% of children in the ceftriaxone group, 15.1% had biliary lithiasis, 5.8% had biliary sludge, and 1.2% had nephrolithiasis. Their study's conclusion was that when considering the use of third-generation cephalosporins, cefotaxime is recommended over ceftriaxone to mitigate the risk of these adverse effects.

Avci et al. conducted a study in which children receiving a seven-day course of standard or high-dose ceftriaxone for various infections exhibited a 7.8% incidence of small renal stones [59]. Notably, previous investigations have typically involved patients with diverse underlying medical conditions. However, this study exclusively focused on patients with pyelonephritis who received ceftriaxone monotherapy. Recent investigations have unveiled a noteworthy occurrence of ceftriaxone-associated urolithiasis. Several cases have been documented involving ceftriaxone treatment leading to various forms of nephrolithiasis in pediatric patients [60-63]. 

It is well-established that biliary pseudolithiasis is a recognized adverse effect associated with ceftriaxone treatment. A prospective study revealed the occurrence of biliary pseudolithiasis in 25-45% of patients subjected to ceftriaxone treatment at dosages ranging from 60 to 100 mg/kg/day. Approximately 33-67% of ceftriaxone is excreted by the kidneys, while the remainder is eliminated via the biliary system [59]. The underlying mechanism responsible for ceftriaxone-associated gallbladder sludge formation has been ascribed to its metabolism and excretion. Ceftriaxone's concentration within the gallbladder can reach levels 20-150 times higher than its serum concentration. The excretion of ceftriaxone has the potential to disrupt the normal excretion of bile acids [64]. This altered milieu within the gallbladder leads to an elevation in the concentration of ionized calcium within the bile. Consequently, ceftriaxone has the propensity to form precipitates with calcium ions, akin to the process involving bilirubin. Consequently, the predominant constituent of ceftriaxone-associated biliary sludge primarily comprises a complex formed between calcium and ceftriaxone. Furthermore, it is worth noting that in an in-vitro study, ceftriaxone itself also influenced the contractile activity of the gallbladder [65]. The principal risk factors predisposing individuals to ceftriaxone-associated GB sludge or stone formation include the administration of high daily dosages (exceeding 2 grams daily), the prolonged duration of drug therapy, and in patients with impaired gallbladder emptying [66,67].

Recommendations by Guidelines

According to the WHO, cefotaxime is the first choice of drug for acute bacterial meningitis, community-acquired pneumonia (severe), complicated intraabdominal infections (mild to moderate), complicated intraabdominal infections (severe), hospital-acquired pneumonia, and pyelonephritis (severe) [68]. Cefotaxime is the second choice of drug for bone and joint infection, pyelonephritis (mild to moderate), and sepsis in neonates, and children. This is consistent with the findings of this review, which indicate that cefotaxime's efficacy in managing severe infections, coupled with its pharmacokinetic profile that enables effective penetration into the central nervous system and other vital sites, makes it particularly well-suited for infections requiring rapid bactericidal activity.

Furthermore, the National Centre for Disease Control, under the purview of the Government of India, has recommended cefotaxime as an empirical and first-line therapy for the treatment of various severe infections [69]. This endorsement has contributed significantly to the extensive utilization of cefotaxime in the country's healthcare landscape. These recommendations are founded on cefotaxime's extensive antimicrobial coverage and favorable safety profile, making it suitable for a wide range of patient groups, including pediatric and critically ill individuals. This aligns with the findings of the review, which indicate that cefotaxime may offer advantages over ceftriaxone in specific clinical situations where its distinct pharmacokinetic properties provide therapeutic benefits.

The reviewed studies demonstrate both benefits and limitations, with variability in designs, patient populations, dosing regimens, and clinical settings, which complicates direct comparisons. Differences in methodologies, such as blinding techniques, sample sizes, and settings (ICU versus general wards), affect the interpretability and generalizability of the findings. However, evidence suggests that cefotaxime may provide advantages in treating severe chest infections and achieving better bacteriologic outcomes in critically ill patients, while ceftriaxone's once-daily dosing offers practical convenience in many settings. Both antibiotics are effective, but their selection should be guided by clinical context, patient-specific factors, and local resistance patterns, optimizing the use of cefotaxime for rapid bactericidal needs and a broader spectrum, and ceftriaxone for its convenience and pharmacokinetic properties.

## Conclusions

Ceftriaxone and cefotaxime exhibit a congruent antibacterial spectrum and analogous therapeutic indications; however, they diverge in terms of their pharmacokinetic attributes. This comprehensive review of the pharmacokinetic profile, safety, and efficacy of cefotaxime compared to ceftriaxone suggests that cefotaxime may be the preferred choice for empirical treatment in patients with similar clinical presentations.
